# Correction: One-pot syntheses of irida-polycyclic aromatic hydrocarbons

**DOI:** 10.1039/c9sc90268f

**Published:** 2019-12-19

**Authors:** Yu Xuan Hu, Jing Zhang, Xiaoyan Wang, Zhengyu Lu, Fangfang Zhang, Xiaofei Yang, Zhihua Ma, Jun Yin, Haiping Xia, Sheng Hua Liu

**Affiliations:** Key Laboratory of Pesticide and Chemical Biology, Ministry of Education, College of Chemistry, Central China Normal University Wuhan 430079 P. R. China yinj@mail.ccnu.edu.cn; Department of Chemistry, Shenzhen Grubbs Institute, Southern University of Science and Technology Shenzhen 518055 P. R. China

## Abstract

Correction for ‘One-pot syntheses of irida-polycyclic aromatic hydrocarbons’ by Yu Xuan Hu *et al.*, *Chem. Sci.*, 2019, **10**, 10894–10899.

The authors regret that there is an ambiguous statement about the synthetic route on Page 1 and would like to clarify the origin of the synthetic route to be entirely clear to readers. Please note that this error does not affect the interpretation of the results. The following statement was added to the front of “In 2018,”. The authors reference work by the Bolaño group, which is reference 14 in the original manuscript, and is included as reference 1 below.

“The synthetic process involved in two step reactions: the first step is the synthesis of methoxy(alkenyl)carbeneiridiums by treating [IrCp*Cl(NCMe)(PMe_3_)]PF_6_ and diarylpropargyl alcohols as demonstrated by the Bolaño group,^1^ and the next step is the synthesis of iridanaphthalenes by reaction of methoxy(alkenyl)carbeneiridiums with silver hexafluorophosphate”.

The Royal Society of Chemistry apologises for these errors and any consequent inconvenience to authors and readers.
